# Adoption and use of immunotherapy in breast cancer management in Africa: barriers and prospect – a narrative review

**DOI:** 10.1097/MS9.0000000000001398

**Published:** 2023-10-12

**Authors:** Jimoh Mutiu Alani, Damilola Quazeem Olaoye, Abdus-Salam Adesina Abass

**Affiliations:** a Radiation Oncology Department, College of Medicine; b Faculty of Pharmacy, University of Ibadan; c University College Hospital, Ibadan, Nigeria

**Keywords:** Africa, breast cancer, cancer, immunotherapy

## Abstract

Breast cancer (BC) is the world’s most frequently diagnosed cancer in women, with 7.8 million women diagnosed with BC in the past 5 years. BC has the highest incidence rate of all cancers in women worldwide (1.67 million), accounting for over 500 000 deaths annually. In Africa, BC accounts for 28% of all cancers and 20% of all cancer deaths in women. The African continent has recorded an alarming increase in incidence, with the highest mortality rate globally. Despite BC being a major health concern in Africa, there is limited access to adequate healthcare services to combat the growing need. Immunotherapy, a promising treatment approach that harnesses the immune system’s power to fight cancer, has shown great potential in BC management. However, in the face of the growing body of evidence supporting its effectiveness, the adoption and use of immunotherapy in BC management in Africa remain limited. Hence, this review aimed to explore the barriers and prospects of immunotherapy adoption and use in BC management in Africa. A comprehensive search across various databases and sources using specific keywords related to immunotherapy and BC to achieve the study aim was conducted. The criteria for including data in the study were based on relevance and availability in English, with no publication year restrictions. The collected data underwent narrative analysis, supplemented by information from sources like country reports, newsletters, commentaries, policy briefs, and direct Google searches. By identifying the challenges and opportunities, this review provided insights into how healthcare providers, policymakers, and other stakeholders can work together to improve the availability and accessibility of immunotherapy to BC patients in Africa.

## Introduction

Highlights

- Breast cancer poses a significant public health challenge in Africa.
- The potential of immunotherapy in its management offers hope.
- Nevertheless, the adoption and use of immunotherapy face obstacles.
- Fortunately, strategies exist to bridge these gaps in low-resource settings.

Although, Africa suffers from a double burden of infectious disease and non-communicable diseases^1^, non-communicable diseases, including breast cancer (BC), are responsible for more than 60% of deaths around the world, with more than 80% of these deaths occurring in low- and middle-income countries (LMICs)^2^. Moreover, ~20% of non-communicable disease deaths are cancer-related. BC is the world’s most frequently diagnosed cancer in women, with 7.8 million women alive diagnosed with BC in the past 5 years^3^. BC has the highest incidence rate of all cancers in women worldwide (1.67 million), accounting for over 500 000 deaths annually^2^. In Africa, BC accounts for 28% of all cancers and 20% of all cancer deaths in women. The African continent has recorded an alarming increase in incidence, with the highest mortality rate globally^4^.

Interestingly, incidence rates are still generally low in Africa, estimated below 35 per 100 000 women in most countries (compared to over 90–120 per 100 000 in North America or Europe)^5^. However, precise data on the incidence figures of BC in Africa are lacking due to the inadequacy of the use or implementation of cancer registries. Despite this, recent incidence data from registries in some African countries like Kampala, the Gambia, and Mali-Bamako provide significant evidence for the popular idea that there is an increase in BC incidence in sub-Saharan Africa^6^. This data evidence on incidence compensates for the increase in mortality recorded in Africa.

The inadequacy of incidence data on BC has not only been caused by systematic healthcare inadequacy in sub-Saharan Africa. The impact of sociocultural beliefs in creating BC-related stigma has also been elucidated in different articles^7^. The gory and fear ascribed to mastectomy has affected the early health-seeking behaviour of people in Africa, which is often worsened by ignorance about the disease, unavailability of tests, and inaccessibility of treatment and detection facilities in this region^8^.

On the other hand, there are standardised BC treatment guidelines that are globally acceptable and adopted by all countries. These guidelines have suggested that the primary choice of therapy should be influenced by cancer staging at diagnosis and receptor expression, for which curative treatment involves some combination of surgery, radiotherapy, and drugs^9^. Due to the peculiarities of developing a standard cancer therapeutics centre in LMICS, the National Comprehensive Cancer Network (NCCN) provided a resource-stratified oncology care guideline to cater for factors that may impede access or acceptance of therapy^10^. Despite this, proper management of cancer patients in developing countries has continued to be a concern as huge gaps have been identified in the quality of care and ease of access to care.

Immunotherapy, a promising treatment approach that harnesses the immune system’s power to fight cancer, has shown great potential in BC management^11^. However, despite the growing body of evidence supporting its effectiveness, immunotherapy’s adoption and use in Africa’s BC management remain limited. Furthermore, the availability of new immunotherapies and the distribution of use in LMICs has been limited due to a variety of reasons, including cost^12^. This review aims to explore the barriers and prospects of immunotherapy adoption and use in BC management in Africa. By identifying the challenges and opportunities, this review seeks to provide insights into how healthcare providers, policymakers, and other stakeholders can work together to improve the availability and accessibility of immunotherapy to BC patients in Africa.

## Methods and materials

We conducted a narrative review of journals using specific keywords to identify the barriers and prospects of the adoption and use of immunotherapy in BC management in Africa. This narrative review was conducted to provide insights into how healthcare providers, policymakers, and other stakeholders can work together to improve the availability and accessibility of immunotherapy to BC patients in Africa. We also believe that this independent review of the level of adoption and the use of immunotherapy will provide insights into practices across Africa and forge ways for inclusion in cancer therapy.

A comprehensive search in Medline, PubMed, PubMed Central, and Google Scholar using search terms including ‘immunotherapy’, ‘Breast Cancer’, ‘immunotherapeutic agents’, ‘Antibody-Drug Conjugates’, ‘early detection’, ‘Immune Checkpoint Blockade’, ‘Triple-Negative’, ‘HER-2’, ‘monoclonal antibody’, ‘low and middle-income countries’ was conducted.

The criteria for including data in this study were as follows: the data sources had to provide information on the adoption and barriers to using immunotherapy in African countries, particularly for BC, and they had to be published in English. There was no restriction on the publication years because there was a lack of available data. Any data sources that did not provide information on immunotherapy use for BC in Africa were excluded.

Two skilled health systems researchers independently conducted a literature review to collect data for the study. The collected articles were managed using Endnote Reference Manager Software version X8, and a preliminary evaluation of the title and abstract was performed to screen for relevance. In cases of duplicate references or disagreements, a consensus was reached through discussion.

The data obtained through the systematic search were subjected to a narrative analysis in order to investigate the study’s objective. In addition, supplementary data were collected from various sources such as country reports, newsletters, commentaries, policy briefs, and other reports. A direct Google search was also conducted to gather relevant information. This approach was taken because it was anticipated that certain valuable sources, such as policy papers, may not have been published in peer-reviewed academic journals due to their non-empirical nature. The extracted data were then discussed in a narrative manner to explore the aim of the study further.

### Immunotherapies in triple-negative and HER2-positive BCs

**Figure 1:**
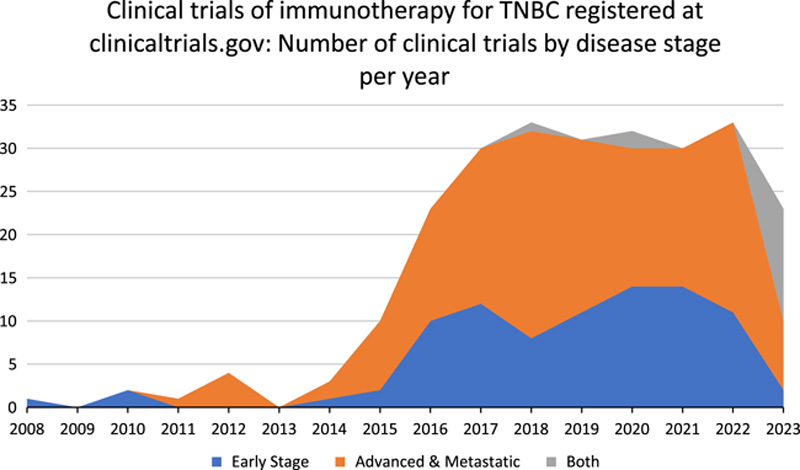
Clinical trials of immunotherapy for TNBC registered at clinicaltrials.gov: number of clinical trials by disease stage per year. TNBC, triple-negative breast cancer.

Triple-negative breast cancer (TNBC) is very complex, heterogeneous, and has restricted treatment options^13^. TNBC, a subtype of BC that is negative for hormone receptors and HER2 (human epidermal growth factor receptor 2), accounts for 10–15% of all BCs^14^. TNBC is also associated with poor prognosis and limited treatment options, progressing quickly and having a high recurrence rate. However, immunotherapy has presented itself as a suitable alternative to TNBC^13^. The recent and novel immunotherapeutic agents have improved the standard of care for TNBC. Immunotherapy has shown great promise in TNBC, with immune checkpoint inhibitors such as pembrolizumab and atezolizumab improving survival in metastatic TNBC^15^. According to Valencia *et al*.^13^, various immunotherapeutic agents are approved for TNBC treatment. As seen in Figure 1, based on different disease stages, the number of clinical trials of immunotherapy for TNBC registered at clinicaltrials.gov has risen over the years. Some of the approved immunotherapeutic agents have included:

1. Atezolizumab is a humanised monoclonal antibody that binds to PD-L1, preventing interaction between PD-1 and B7. It is often combined with paclitaxel and chemotherapy for TNBC treatment^15^.
2. Pembrolizumab has shown effectiveness in shrinking TNBC tumours and managing toxic side effects of cancer. It is also often used in combination with chemotherapy and neoadjuvant chemotherapy^15^.
3. Durvalumab: A PD-L1 inhibitor, has been demonstrated by the GeparNUEVO trial to significantly improve long-term survival when added to neoadjuvant chemotherapy in the management of TNBC^16^.
4. Vaccines: Vaccines that target HER2-derived peptides are currently being investigated. Other non-HER2 tumour-associated antigens being targeted by vaccines include cancer testis antigen and Mucin 1^17^.
5. Adoptive cell therapy, which includes tumour-infiltrating leucocyte-based therapies, T-cell receptor gene therapy, and CAR (chimeric antigen receptor) T-cell therapy, is also being evaluated^17^.

There is promising evidence of the use of immunotherapy in HER2-positive BC treatment, and this represents a fascinating field that is still currently under investigation^18^. As seen in Table 1, there continue to be clinical trials for the treatment of HER2-positive tumours, including breast tumours. Monoclonal antibodies, antibody–drug conjugates, immune checkpoint inhibitors, CAR T cells, and vaccines are immunotherapies employed in HER2-positive BC treatment^18^. Monoclonal antibodies such as trastuzumab and pertuzumab activate the immune system, creating a direct anti-tumour effect.

**Table 1:** HER2 bispecific antibodies in clinical trials for the treatment of HER2-positive tumours, including breast tumours.

NCT number	Drug	Target antigens	Status	Sponsor/collaborators	Phases	Start
NCT02829372	GBR 1302	HER2 × CD3	Terminated	Ichnos Sciences SA|Glenmark Pharmaceuticals S.A., La Chaux-de-Fonds, Switzerland	Phase 1	May 2016
NCT03983395	ISB 1302	HER2 × CD3	Terminated	Ichnos Sciences SA|Glenmark Pharmaceuticals S.A., La Chaux-de-Fonds, Switzerland	Phase 1|Phase 2	Apr 2020
NCT05076591	IMM2902	HER2 × CD47	Recruiting	ImmuneOnco Biopharmaceuticals Inc., Shanghai, China	Phase 1	Jun 2022
NCT04162327	IBI315	HER2 × PD-1	Recruiting	Innovent Biologics Co., Ltd., Suzhou, China	Phase 1	Nov 2019
NCT03650348	PRS-343	HER2 × 41BB	Active, not recruiting	Pieris Pharmaceuticals, Inc., Boston, MA, USA	Phase 1	Aug 2018
NCT03330561	PRS-343	HER2 × 41BB	Completed	Pieris Pharmaceuticals, Inc., Boston, MA, USA	Phase 1	Sep 2017
NCT05523947	YH32367	HER2 × 41BB	Recruiting	Yuhan Corporation, Seoul, Korea	Phase 1|Phase 2	Aug 2022

Source: Duro-Sánchez S, *et al*.^56^.

Antibody–drug conjugates are monoclonal antibodies attached to cytotoxic drugs, and this enables the delivery of cytotoxic drugs to the tumour sites directly; examples of these conjugates are trastuzumab–emtansine, trastuzumab–deruxtecan, and trastuzumab duocarmazine^19^. Immune checkpoint inhibitors help to deal with the resistance developed from using monoclonal antibodies and conjugate immunotherapies. CAR T cells use specific target antibody action and T cells to project anti-tumour activity in HER2-positive cancers. According to Agistinetto *et al*.^18^, cancer vaccines target immunogenic peptides HER2 tumours. These vaccines also stimulate T cells to recognise and destroy HER2 cancer cells.

### Estrogen receptor-positive BC

Estrogen receptor (ER)-positive BCs are BCs that are positive for estrogen receptors and negative for HER2^20^. They are considered one of the most common classes of BCs that occur in women, representing about 65% of cases of BC in women who are less than 50 years of age and 75% of BC cases in women above 50 years of age^21^. In Africa, reports about hormone receptor and molecular subsets of BC are contradictory. Some reports state that breast tumours in African women are hormone receptor-negative, while other reports show the prevalence of hormone receptor-positive breast tumours^22^. According to Abramson Cancer Center (n.d.), estrogen-positive cancer cells can receive signals from the hormone estrogen that instructs the cells to grow.

ERs are nuclear proteins that regulate the expression of specific genes that control cell growth^23^. The estrogen hormone binds to the ER, which stimulates receptor-regulated transcription and promotes the growth and proliferation of tumour cells^20,23^. ER-positive BC is often associated with heterogeneity, and tumours have varying levels of ER and progesterone receptor expressions.

The degree of growth of the tumour cells is, however, driven by estrogen receptor, which also controls the form of genetic expressions and type of genetic mutations. About 10% of ER-positive cancers are hereditary, and such genes include CHEK2, BRCA1, BRCA2, ATM, and PALB2^20^. According to Kelsey *et al*.^23^, various factors related to reproduction affect BC risks due to alterations in exposures to ovarian hormones and exogenous hormones, such as early menarche, late menopause, late pregnancies, hormone replacement therapy, and hormonal contraception.

According to Burstein^20^, modes of treatment for estrogen receptor-positive BC include adjuvant hormone therapy, which is recommended in patients for about 5–10 years, chemotherapy, neoadjuvant therapy, targeted therapies using cyclin-dependent kinases 4/6, and surgery. The most common procedure in applying hormone therapy in BC treatment is the use of selective estrogen receptor modulators (SERMs). The mechanism of action of these modulators is to bind to hormone estrogen receptors, which in turn prevent the proliferation of tumour cells through estrogen^20^. Tamoxifen is a SERM famously used for the treatment of ER-positive BC. It is also used in combination with other BC treatment therapies and has had great success rates. According to Carleton *et al*.^24^, to improve treatment for ER-positive BC patients, periodic screening, genetic screening, pathological determination of recurrence, radiation therapy, and breast surgery adjuvant endocrine therapy are clinical stages of treatment that should be implemented.

### Use of immunotherapy and barrier to use in Africa

Immunotherapy is a targeted therapeutic approach that focuses on modifying the mechanisms of the immune system to combat various diseases. This is an essential and indispensable cancer treatment strategy with novel T-cell targeting agents approved by the FDA across various cancer subsets, both as single agents and combination cytotoxic agents^25^. While immunotherapy has been shown to be effective in HER2-positive and TNBC subtypes^18^, equitable access to these drugs has been proven to be important in achieving good BC prognosis. Despite these promises, a number of growing evidences have reported several barriers to the use and access of these immunotherapeutic agents^26–28^.

Furthermore, BC in Africa accounts for 28% of all cancers and 20% of all cancer deaths in women. Yet, according to Sharma *et al*.^25^, Africans make up only one-sixth of the global population involved in immunotherapy clinical trials, although less than 1% are conducted on the African continent. Also, these limited clinical trials (which form the basis of guidelines/recommendations) in Africa lead to a paucity of data on the efficacy/response to treatment following immunotherapy for BC in Africans residing in Africa, as most times, data for Africa are extrapolated from data from Africans outside of Africa. This could also account for the reluctance to use immunotherapy in Africa^25^.

Disappointingly, clinical trials have also not yet demonstrated a synergistic effect of immunotherapy and HER2-targeting agents. Studies examining the potential of immune checkpoint inhibitors in metastatic HER2-positive BC patients have yielded low anti-tumour efficacy in unselected, heavily pretreated patients, with limited activity mostly being seen in PD-L1-positive tumours^29–31^. The early stages of cancer, which is usually confined to the breast, involve a less invasive tumour and a more permissive microenvironment allowing the immune system to recognise and respond to tumour antigens. On the contrary, advanced tumours have a higher tumour burden, resistant cells, and an immune-tolerant microenvironment, and metastatic patients tend to be systemically immunosuppressed. Therefore, investigating immunotherapy in the early setting for HER2-positive BC is a promising strategy^18^.

Trastuzumab and pertuzumab have been established to be effective in early-stage, HER2-positive BC patients, as well as in situations of advanced metastasis^32^. Recently, immune checkpoint inhibitors such as pembrolizumab and atezolizumab have been effective in HER2-positive BC^14,15,33^.

Despite these prospects, the use of immunotherapy is very limited in developing countries in Africa because of unavailability, financial burden, and lack of unifying guidelines for use^34^. Many immunotherapy drugs are not available in Africa, and those available are often very expensive. In addition, there are barely any standard healthcare facilities with regulatory requirements to administer these medications. According to Akl *et al*.^12^, another barrier to use is the limited number of oncology professionals available in Africa to deal with the side effects of novel immunotherapies. Other barriers to adopting immunotherapy for BC in Africa include limited awareness and education about immunotherapy among healthcare professionals and patients, contributing to limited adoption and use of immunotherapy in Africa^35^. Most healthcare professionals in Africa lack the necessary knowledge and skills to administer and manage immunotherapy, and patients are often unaware of its potential benefits. Also, the lack of clear regulatory frameworks for immunotherapy in Africa contributes to limited adoption and use. Most African countries lack clear guidelines and regulations for immunotherapy administration and management, making it difficult for healthcare professionals to adopt and use immunotherapy. International guidelines which have included immunotherapy for BC are available, and some have been harmonised for use in Africa. However, there are some barriers to adopting these guidelines, such as being generalised, oversimplified, or complicated; concerns about the evidence that forms the basis of the guidelines; and clinicians’ negative perceptions of the guidelines^35^.

With the increasing complexity of cancer care and the demand on healthcare providers, the prospect of getting access or use of immunotherapy has been hinged on several organisational policies and factors. The geographic location of healthcare facilities will play a huge role in the socioeconomic class of patients around them and, in turn, determine the spending capabilities of these patients on healthcare needs. The ease of referral and appointment systems is a huge factor that determines the level of satisfaction patients get from healthcare services. The traditional process of scheduling outpatient centres by walk-in has led to long waiting times in the queue to receive services^36^. Several pieces of evidence have shown that non-user friendly appointment system is the main barrier to patients’ medical follow-up compliance^37^. The increasing diversity of African nations continues to precipitate challenges for healthcare system policymakers in making policies that create and deliver dynamic yet culturally competent services. The availability of culturally competent services and healthcare worker diversity have proven to influence patients’ retention in healthcare facilities. Nair *et al*.^38^ discussed the impacts of Social Determinants of Health (SDH) on racial and minority groups, which in turn limit the willingness of these groups to seek healthcare services. Kutalek^39^ posited that differences in healthcare treatment and outcomes among minorities persist even after adjusting for socioeconomic factors.

Affiliation of healthcare facilities with research or academic systems has been shown to influence the access to clinical trials^40^. Osarogiagbon *et al*.^41^ reported that about 75% of patients seeking cancer are unable to enrol in clinical trials because the institutions where they seek care do not have appropriate clinical trials available for them or, when available, the eligibility criteria prevent enrolment.

Although BC has been challenging to treat with immunotherapies, studies have shown that immunotherapy can potentially improve the outcomes of subsets of BC^40^. The improved technologies in immunotherapeutic treatments have impacted dynamic changes in BC treatment. This advancement will result in the clinical success of immunotherapies^40^. Therefore, with increased access to immunotherapeutic drugs and provided healthcare coverage for BC patients, the future of immunotherapeutic therapy can be promising in Africa.

### Bridging barrier

More than ever before, the need to bridge barriers to equitable access to cancer therapy, in this case immunotherapy, has been a cause of concern globally. Several efforts have been put into stratifying and classifying the global barriers and potential solutions. As seen in Table 2, different contextual solutions have been identified to the barriers to immunotherapy use in Africa. According to Osarogiagbon *et al*.^41^, the barriers and solutions to equitable access can be stratified into Patient, Provider, Health care, and Societal levels. Several potential solutions can be explored to overcome the barriers to adopting immunotherapy in BC management in Africa. Due to the high cost of immunotherapy and, on a wider scale, cancer therapy, especially in a low-resource setting like Africa, equitable access to health insurance coverage is a crucial factor that reduces the financial toxicity induced by cancer care and thereby increases active involvement in clinical trials^42–44^. However, it is essential to acknowledge the fact that assurance of health insurance coverage does not guarantee comprehensive access to cancer treatments or opportunities for participation in clinical trials^45^. The reasons for these are well-founded in other sociocultural factors that may impede the healthcare-seeking behaviour of patients^46^. To bridge this barrier, improving patient understanding and dispelling myths through educational initiatives that will facilitate efficient communication between patients and healthcare providers has become imperative. This communication should be all-encompassing, including disease-related information, patients and provider expectations, the healthcare system, available treatment options, associated costs, opportunities for cost alleviation or subsidies, the importance of patients’ adherence to guidelines and treatment schedules, and potential benefits and adverse effects of drugs^47^. Several pieces of evidence have shown that involving patients in decision-making processes concerning their healthcare also improves their overall response to treatment and participation in trials^47,48^. In addition, the involvement of Africa in these clinical trials helps to advance cancer treatment therapy for patients in Africa because it introduces them to novel therapy and provides training and experience for healthcare providers in the unique toxicity profile of immunotherapeutic molecules^25^. To supplement this, there is a need to increase awareness and knowledge of immunotherapy among healthcare providers in Africa. This can be achieved through training programmes, workshops, and conferences that provide healthcare providers with the necessary skills and knowledge to administer immunotherapy correctly.

**Table 2:** Barriers and solutions to barriers to the use and adoption of immunotherapy in Africa.

Barrier	Solutions
Cancer immunotherapy drugs are expensive Exerts economic strain on healthcare system and patient finances	The application of product development partnerships, as it has been done with non-communicable diseases, is to alleviate some costs incurred during production, leading to the eventual reduction of drug prices in the market. Use cost-effectiveness, cost–benefit, and QoL assessments to evaluate the relationship between clinical benefit and treatment cost. Develop novel drug reimbursement modalities.
Non-involvement of Africans in Africa in clinical trials	The involvement of Africa in these clinical trials helps to advance cancer treatment therapy for patients in Africa because it introduces them to novel therapy and provides training and experience for healthcare providers in the unique toxicity profile of the immunotherapeutic molecule. Educate patients, caregivers, and their influencing network on the value of clinical trial participation (the best treatment is a clinical trial); simplify and demystify the clinical trial enrolment process for patients and caregivers.
Lack of clear regulatory frameworks for immunotherapy in Africa Lack of unifying guidelines for use	Through the medical and pharmaceutical councils, clear guidelines on handling and administration of immunotherapy will be instituted to ensure unifying standard of practice.
Limited number of oncology professionals available in Africa to deal with the side effects of novel immunotherapies Knowledge and skills to administer and manage immunotherapy	There is a need to increase awareness and knowledge of immunotherapy among healthcare providers in Africa. This can be achieved through training programmes, workshops, and conferences that provide healthcare providers with the necessary skills and knowledge to administer immunotherapy correctly.
Limited awareness and education about immunotherapy among healthcare professionals and patients Patients are often unaware of its potential benefits	To bridge this barrier, improving patient understanding and dispelling myths through educational initiatives that will facilitate efficient communication between patients and healthcare providers has become imperative.
The geographic location of healthcare facilities	Improve public transportation services; promote telehealth services; leverage information technology to expand patient-level access to care.
The availability of culturally competent services and healthcare worker diversity	Improving the cultural competency of oncologists and related medical professionals as it relates to treating cancer patients is pivotal to enhancing the diagnosis, practice, and management of cancers. In the African landscape, understanding cultural practices, ethical nuances, and boundaries would go a long way in improving cancer care delivery, participation in clinical trials, and overall healthcare statistics.

Furthermore, there is a need for key players’ involvement in increasing access to immunotherapy drugs in Africa. This can be achieved through partnerships between African governments, pharmaceutical companies, and international organisations to negotiate lower drug prices and increase the availability of immunotherapy drugs in Africa. Before now, pharmaceutical companies have moved to adopt more innovative models to address the significant challenges drug costs pose, especially in LMICs. As key stakeholders in the drug development process, the pharmaceutical industry carries a significant degree of responsibility in finding solutions. Over the past few decades, the majority of research-based pharmaceutical companies have intensified efforts to ensure access to vital medications in developing nations. This has been accomplished through various means, such as lending support or actively engaging in product development partnerships (PDPs)^49,50^. The application of PDPs, as it has been done with non-communicable diseases, is to alleviate some costs incurred during production, leading to the eventual reduction of drug prices in the market^51^.

Additionally, improving the cultural competency of oncologists and related medical professionals as it relates to treating cancer patients is pivotal to enhancing the diagnosis, practice, and management of cancer. In the African landscape, understanding cultural practices, ethical nuances, and boundaries would go a long way in improving cancer care delivery, participation in clinical trials, and overall healthcare statistics^52,53^. Adequate development of programmes that enlighten healthcare providers from their younger years of practice, recruitment of staff from diverse cultural backgrounds, and proper patient–provider communication lines are critical in improving the competency and understanding of cancer care delivery^54,55^.

## Conclusion

BC is a significant public health concern in Africa, and the use of immunotherapy in its management holds promise. However, the adoption and use of immunotherapy in Africa face several challenges, including the high cost of immunotherapy drugs, lack of awareness and knowledge about immunotherapy among healthcare providers, and limited infrastructure and resources to support immunotherapy. Providentially, several strategies exist to bridge the gaps in the utilisation of novel drugs like immunotherapy, especially in low-resource settings like Africa. Firstly, it is crucial to ensure equitable access to clinical facilities that use immunotherapies as soon as they become approved for adoption and use. Furthermore, given the peculiar economic situation of many Africans, who may particularly benefit from these immunotherapies, the high costs associated with these drugs must be addressed. Addressing financial barriers to treatment and advocating for policies that reduce prices have become imperative.

Without a doubt, addressing these challenges will require holistic collaboration from healthcare providers, policymakers, and other stakeholders to enhance the quality of epidemiological data on BC and improve the availability, accessibility, affordability, and acceptance of immunotherapy in Africa.

## Ethical approval

Ethical approval was not required for this review.

## Consent

Informed consent was not required for this review.

## Sources of funding

This review was sponsored by Pfizer Specialities Limited.

## Author contribution

This review was conceptualised by D.Q.O. and J.M.A. All authors contributed significantly to the article search and writing the article. A.-S.A.A. and J.M.A. further reviewed the first draft of the manuscript. All the authors read and approved the final manuscript.

## Conflicts of interest disclosure

The authors declare no conflicts of interest.

## Research registration unique identifying number (UIN)

Not applicable.

## Guarantor

Dr Jimoh Mutiu.

## Data availability statement

Not applicable.

## Provenance and peer review

Not applicable.
